# A hypothesis for the evolution of the upper layers of the neocortex through co-option of the olfactory cortex developmental program

**DOI:** 10.3389/fnins.2015.00162

**Published:** 2015-05-12

**Authors:** Federico Luzzati

**Affiliations:** ^1^Department of Life Sciences and Systems Biology (DBIOS), University of TurinTurin, Italy; ^2^Neuroscience Institute Cavalieri OttolenghiOrbassano, Truin, Italy

**Keywords:** neocortex evolution, piriform cortex, pallium, upper layers, cell type homology, spatial patterning, temporal patterning, doublecortin

## Abstract

The neocortex is unique to mammals and its evolutionary origin is still highly debated. The neocortex is generated by the dorsal pallium ventricular zone, a germinative domain that in reptiles give rise to the dorsal cortex. Whether this latter allocortical structure contains homologs of all neocortical cell types it is unclear. Recently we described a population of DCX+/Tbr1+ cells that is specifically associated with the layer II of higher order areas of both the neocortex and of the more evolutionary conserved piriform cortex. In a reptile similar cells are present in the layer II of the olfactory cortex and the DVR but not in the dorsal cortex. These data are consistent with the proposal that the reptilian dorsal cortex is homologous only to the deep layers of the neocortex while the upper layers are a mammalian innovation. Based on our observations we extended these ideas by hypothesizing that this innovation was obtained by co-opting a lateral and/or ventral pallium developmental program. Interestingly, an analysis in the Allen brain atlas revealed a striking similarity in gene expression between neocortical layers II/III and piriform cortex. We thus propose a model in which the early neocortical column originated by the superposition of the lateral olfactory and dorsal cortex. This model is consistent with the fossil record and may account not only for the topological position of the neocortex, but also for its basic cytoarchitectural and hodological features. This idea is also consistent with previous hypotheses that the peri-allocortex represents the more ancient neocortical part. The great advances in deciphering the molecular logic of the amniote pallium developmental programs will hopefully enable to directly test our hypotheses in the next future.

## Introduction

The Neocortex is a pallial structure that is divided in multiple sub-regions and is made by six layers of distinct neuronal types. Despite more than a century of intense research and speculation, the evolutionary origin of this brain region is still unclear (Reiner, 2000; Butler et al., 2011; Aboitiz and Zamorano, 2013; Medina et al., 2013). Early work of Karten identified neuronal types in the hyperpallium/dorsal cortex and dorsal ventricular ridge (DVR) of sauropsids that show patterns of connections similar to neurons of the mammalian neocortex (Karten, 1969, 2013; Butler, 1994). In particular the hyperpallium/dorsal cortex has been shown to receive visual and somatosensory information from lemno-thalamic nuclei and may thus be homologous to the visual and somatosensory cortex of mammals, while the DVR receive collo-thalamic auditory and visual projections and may be homologous to regions receiving similar projections in the temporal neocortex (Karten, 1969, 2013; Desan, 1984; Butler, 1994; Butler et al., 2011).

**Figure 1:**
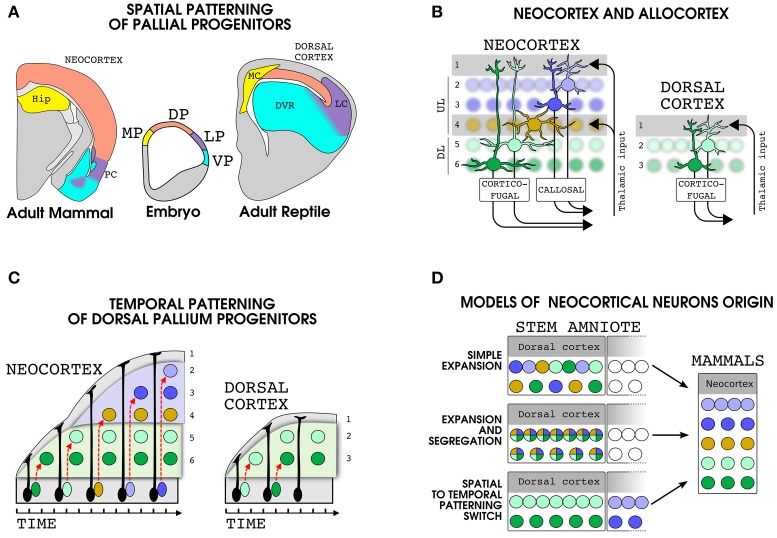
**Development and evolution of dorsal pallial derivatives in amniotes. (A)** Spatial patterning mechanisms subdivide the embryonic pallial progenitors in at least four domains: Medial pallium (MP, yellow), dorsal pallium (DP, pink), lateral pallium (LP, violet) and ventral pallium (VP, cyan). The brain regions produced by each domain are shown in a mammal (left) and a reptile (right) with the same color code. **(B)** Schematic representation of the organization of the cellular layers and main input and output projections in the neocortex (left) and in the reptilian dorsal cortex (right). **(C)** Development of dorsal pallium glutamatergic neurons in mammals and reptiles. In the neocortex the same progenitor produces multiple cell types in an inside-out sequence. In the reptilian dorsal cortex, the dorsal pallium progenitors produce a reduced number of cells in an outside-in sequence. The migration of immature neuroblasts is indicated by a dashed red arrows. Please note that, along with our theory, in **(B,C)** we used the same color code for the mammalian DL and reptilian dorsal cortex neurons. Nonetheless, it is important to note that the precise correspondence between layer II and III neurons of the dorsal cortex and layer V and VI of the neocortex is not known. **(D)** Models of the evolution of the principal neurons of the neocortex. Each color represent a set of gene modules specifying a particular neuronal types in the modern neocortex (right). Three different possibile pattern of expression of these gene modules in cells of the dorsal cortex of the stem amniote are shown (left). Abbreviations: HIP, hippocampus; MC, medial cortec; LC, lateral cortex; PC, piriform cortex.

Nonetheless, subsequent work showed that during early development pallial progenitors of all tetrapods are regionalized into at least four conserved domains, referred as medial (MP), dorsal (DP), lateral (LP), and ventral (VP) pallium, that give rise to distinct radially migrating glutamatergic neurons (Fernandez et al., 1998; Puelles et al., 2000; Brox et al., 2004). The neocortex is generated by DP progenitors that in sauropsids give rise only to the hyperpallium (in birds) and the dorsal cortex (in reptiles; Figure 1A). By contrast the DVR is generated by LP and VP progenitors that in mammals give rise to claustro-amygdalar nuclei together with structurally and functionally conserved regions receiving olfactory and pheromonal information (olfactory cortex and cortical/medial amygdala respectively). These studies strongly suggests that the neocortex is homologous, as a field, only to the hyperpallium/dorsal cortex while the DVR is homologous to the amygdala, that also receives auditory and collo-thalamic visual projections, the claustrum and the entopeduncular nucleus (Bruce and Neary, 1995; Striedter, 1997; Puelles et al., 2000; Puelles, 2001; Butler and Molnár, 2002; Bruce, 2007; Medina et al., 2013).

## Organization of dorsal pallial derivatives in mammals and reptiles

It is generally accepted that in the reptilian ancestor of mammals the medial, dorsal and lateral cortices were laminated but were made only by three layers, an organization that is also called allocortex (Figure 1B; Nieuwenhuys, 1994; Reiner, 2000; Shepherd, 2011; Fournier et al., 2015). In mammals, this type of cortex is still present in two structurally and functionally well conserved regions that border the neocortex: the hippocampus, a MP derivative, and the piriform cortex, a LP derivative that receive a direct input from the olfactory bulb. In the allocortex the more superficial layer I is a plexiform layer where extrinsic and intrinsic projections meet the apical dendrites of pyramidal neurons whose cell bodies settle in layers II and III (Haberly, 1990; Ulinsky, 1990). In general, the cellular density is higher in layer II than in layer III particularly in the piriform/lateral cortex and the hippocampus. The neocortex shares with allocortex the basic microcircuits, but it stands out for the higher number of neurons and layers (Shepherd, 2011; Fournier et al., 2015). In many respects the neocortex can be described as a double allocortex, with two couples of pyramidal layers, namely upper (II,III,IV; UL) and deeper (V,VI, DL), each below a plexiform layer carrying extrinsic inputs, namely layer I and IV (Figure 1B; Shepherd, 2011). In primary sensory areas the layer IV is enriched in stellate cells, a glutamatergic cell type that lack apical tufts and output projections and is specialized in receiving thalamic inputs (Sanides, 1969; Jones, 1975). By contrast, most of the glutamatergic neurons in the other layers possess an apical dendrite directed to layer I and output connections emerging at the opposite pole of the cell body (Figure 1B). The UL neurons axons are mainly involved in cortico-cortical connectivity and include homotopic and heterotopic callosal projections to the contralateral hemisphere, while DL neurons target various subcortical structures (Figure 1B; Shepherd, 2011; Greig et al., 2013). To understand the evolution of the neocortex we should thus first disclose the developmental mechanisms that triggered the multiplication of cellular and plexiform layers. As expected, comparative studies shows that in respect to reptiles, the mammalian DP progenitors have an increased proliferation (Nomura et al., 2013, 2014) that include the appearance of a well defined layer of intermediate progenitor cells: the SVZ (Martínez-Cerdeño et al., 2006; Abdel-Mannan et al., 2008; Cheung et al., 2010). In mammals, this increase in cell proliferation is accompanied by a distinct pattern of migration of neuroblasts that passes older cells (n.b. in both piriform cortex and neocortex; Bayer, 1986) rather than accumulating below them as in reptiles (Goffinet et al., 1986; Figure 1C). Since cortical neurons are generally considered to be already committed to a specific cell type at their birth (Greig et al., 2013; Rouaux and Arlotta, 2013), a major point to understand the emergence of the neocortex will be to unravel the evolution of the developmental program set up by dorsal pallium progenitors and regulating the production of neocortical glutamatergic neurons.

## Models of transition from a three to a six layered cortex

The study of the organization of genes underlying cell identity suggests that genes sub-serving specific functions can be grouped into modules whose expression is regulated by a limited number of transcription factors also called “selector genes” (Arendt, 2008; Achim and Arendt, 2014). In this model, during development morphogens regulate patterning by inducing the expression of the selector genes at specific times and place. Starting from these considerations, three major mechanisms have been recently proposed to underlie the evolution of new cell types from a precursor cell in a given lineage: (1) *Divergence of functions*, in which two sister cell types inherit the same gene modules and gradually modify them with time, (2) *Segregation of functions*, in which two sister cell types lose complementary parts of the gene modules of the former precursor cells. (3) *Co-option of functions*, in which the precursor cell co-opts the gene modules of an unrelated cell type (Arendt, 2008; Achim and Arendt, 2014). It is to note that the term co-option generally refers to the acquisition of new roles by pre-existing characters (True and Carroll, 2002). In the specific case of the gene regulatory networks controlling cell type specification, co-option may occur for cis- and trans- acting transcriptional regulators at multiple levels and can thus be involved in all the presented modes of cell type evolution. Nonetheless, for the *co-option of functions* hypothesis these mechanisms should act at the level of selector genes, thus leading to the ectopic expression of the pre-existent gene regulatory networks of complex developmental programs. This latter possibility has been proposed to explain multiple evolutionary innovations such as the evolution of novel sex determining genes (Sutton et al., 2011; Takehana et al., 2014) or the acquisition of a chondrogenic fate in the neural crest lineage (Meulemans and Bronner-Fraser, 2007; Hall and Gillis, 2013).

The specification of neocortical neurons depends on spatial patterning events delimiting the DP progenitors (Figure 1A; see for review Puelles, 2011), followed by temporal patterning mechanisms that lead these cells to sequentially produce the DL (first) and UL (last) (Figure 1C; Angevine and Sidman, 1961; Greig et al., 2013; Gao et al., 2014). When applying the above mentioned concepts to the evolution of the neocortical neurons, three main hypotheses can be drawn (Figure 1D): (1) *Simple Expansion*: DP progenitors of the reptilian ancestors produced homologous of both UL and DL neocortical neurons following the same temporal patterning mechanisms as in the modern neocortex. In this model the emergence of the neocortex was driven by changes only in the proliferation of DP progenitors and migration of their daughter cells. (2) *Expansion and Segregation*: gene modules underlying specific functions of UL and DL were present in a single precursor cell in the ancestral DP derivatives and became segregated and subsequently refined in distinct sister cell types. In this case the temporal patterning of DP progenitors will be a mammalian innovation. (3) *Spatial to Temporal patterning switch*: DP progenitors co-opted the expression of gene modules specifying the neuronal types of other pallial regions (i.e., MP, VP or LP), thus leading to the appearance of new cell types in the DP derivatives. The temporal patterning of neocortical progenitors may thus represent a patchwork of formerly spatially segregated developmental programs. In this case part of the neocortical cells may have a sister cell type in a different pallial domain.

Some evidences against the first two hypotheses were first presented by Ebner based on hodological considerations (Ebner, 1976). Indeed, reptilian dorsal cortex neurons have projections to subcortical targets that resemble those of neocortical DL neurons but lack the extensive network of intracortical connections, including homotopic contralateral projections, that are typical of UL neurons (Ebner, 1976; Desan, 1984; Hoogland and Vermeulen-Vanderzee, 1989). Thus, Ebner proposed that the UL neurons may represent an evolutionary novelty. In the early'90 Anton Reiner extended Ebner hypotheses by showing that UL specific interneurons are lacking from the reptilian dorsal cortex (Reiner, 1991, 1993). However, later studies showed that interneurons are generated in the sub-pallium (Cobos et al., 2001; Wichterle et al., 2001) and this makes the Reneir's observations only indirectly related to the DP progenitors developmental program. In 2009 we described a specific population of neurons of the layer II of the neocortex that according with Ebner and Reiner ideas was absent from the dorsal cortex of *Lacerta Muralis*, a lizard. However, virtually identical cell types were observed in the LP and VP derivatives of both lizard and mammals thus supporting the spatial to temporal patterning switch hypothesis (Luzzati et al., 2009). The interest about these cells comes from the fact that (1) they express Tbr1, suggesting a pallial origin, and (2) morphological and distributive features support that they represent a specific neuronal population that is shared by different pallial derivatives and tetrapod species. In the following sections we will describe and discuss in detail our observations in the context of more recent data that further support these hypotheses.

## Old cells in new layers: the strange case of the DCX+ cells in the layer II of different amniote pallial derivatives

**Figure 2:**
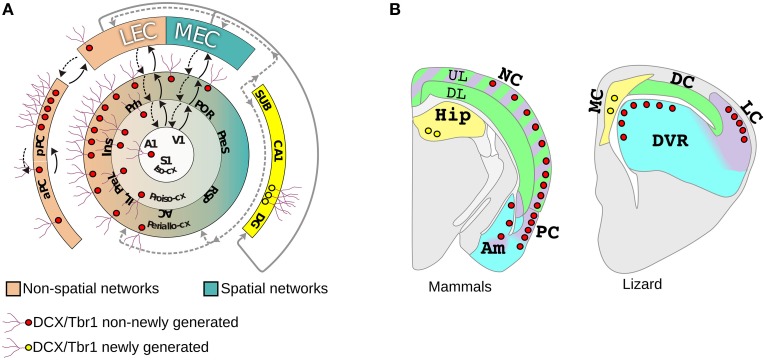
**Distribution of DCX+/Tbr1+cells in the pallium of mammals and a lizard. (A)** The distribution of layer II DCX+/Tbr1+ cells either newly generated (yellow) or non-newly generated (red) is shown in a schematic view of mammalian allocortical and neocortical regions. The neocortex is schematized according to Sanides in three concentric rings: peri-allocortex, proisocortex, and isocortex. The isocortex contains the primary sensory areas from which information flows through a hierarchichal sequence of areas and reaches either the lateral (LEC) or medial enthorinal cortex (MEC). Areas connected with LEC and MEC are shown in pink and turquoise green respectively, feedforward (solid arrows) and feedback (dashed arrow) pathways are also shown. **(B)** Schematic coronal sections showing the distribution of DCX+/Tbr1+ cells in different pallial domains in mammals (left) and lizard (right). Additional abbreviations: A1, primary auditory cortex; AC, anterior cingulate cortex; DP, dorsal pallium; IL, infralimbic cortex; Ins, insular cortex; pPC posterior piriform cortex; aPC; anterior piriform cortex; PreL, prelimbic cortex; PreS, presubiculum; POR, postrhinal cortex; Prh, perirhinal cortex; RSP, retrosplenial cortex; SUB, subiculum. Redrawn from Luzzati et al. (2009).

Doublecortin (DCX) is a microtubule associated protein involved in cytoskeletal dynamics during migration and differentiation of immature neurons (Francis et al., 1999; Gleeson et al., 1999; Friocourt, 2003). Accordingly, in the adult brain the expression of DCX is restricted to regions of ongoing neurogenesis (Nacher et al., 2001; Brown et al., 2003; Couillard-Despres et al., 2005; Luzzati et al., 2006; Balthazart and Ball, 2014). The only clear exception to this rule is a population of neurons in the layer II of the piriform cortex and neocortex (Gómez-Climent et al., 2008; Luzzati et al., 2009) that are not adult generated but show a strong and homogeneous DCX immunoreactivity that closely resembles that of immature neurons. Layer II DCX+ cells occurs in two main morphological subtypes: Type I cells have small cell bodies and dendrites confined to layer II, while type II cells have larger cell bodies and send one or two dendritic branches to layer I (Luzzati et al., 2009). Electrophysiological analyses in DCX-GFP mice piriform cortex revealed that type I cells resemble immature neurons, while most type II cells shows mature features with large Na+ currents and multiple action potentials (Klempin et al., 2011). In both piriform cortex and neocortex type I and II DCX+ cells express Tbr1 suggesting that they are glutamatergic neurons derived from pallial germinative zones (Englund et al., 2005; Hevner et al., 2006; Luzzati et al., 2009). Interestingly the clear predominance of subpial dendrites over basal dendrites place type II cells within the population of atypical pyramidal cells previously defined as “extraverted neurons” (Sanides and Sanides, 1972). Since the lack of basal dendrites represent an ancient feature in the evolution of pyramidal cells, extraverted neurons in the neocortex were originally considered a conserved cell type. Besides laboratory mice and rats (Nacher et al., 2001; Luzzati et al., 2009), in which layer II DCX+ cells are scarce and mostly restricted to the piriform and perirhinal cortices (Nacher et al., 2001), in all other mammalian species analyzed so far such as rabbits (Luzzati et al., 2009), guinea pigs (Xiong et al., 2008; Luzzati et al., 2009), cats (Cai et al., 2009), dogs (De Nevi et al., 2013), giant african mole rats (Olude et al., 2014), epaulatted fruit bats (Gatome et al., 2010), reshus macaques (Cai et al., 2009; Fung et al., 2011), and humans (Cai et al., 2009), DCX+ cells in layer II are abundant and widely distributed in both piriform cortex and neocortex. A detailed analysis of the distribution of these cells in rabbits and guinea pigs revealed that layer II DCX+ cells are specifically associated to the network of brain regions connected to the lateral entorinal cortex (LEC; Figure 2A; Luzzati et al., 2009). These brain regions, including the rostro-lateral neocortex and piriform cortex, receive information about local sensory objects and have been implicated in non-spatial cognition. By contrast caudo-medial neocortical areas connected to the Medial EC (MEC) and processing information of both external and internal stimuli involved in spatial cognition, are mostly negative for DCX (for anatomical and functional descriptions of LEC and MEC connections see Burwell and Amaral, 1998a,b; Jones and Witter, 2007; Knierim et al., 2014). Within LEC connected networks the DCX+ cells show a strong preferential distribution in higher order areas such as posterior piriform cortex, secondary sensory areas, insular, perirhinal cortex and prefrontal cortex (Figure 2A). Altogether, the similarities in the morphology, laminar position and preferential distribution in higher order areas strongly suggests that DCX+ cells of the neocortex and piriform cortex may represent a common cell type that is shared by these two regions.

Notably, in the lizard *L. Muralis* we identified DCX+/Tbr1+ cells morphologically similar to those of mammals in the layer II of the olfactory cortex and DVR, with a preferential distribution in higher order areas, but not in the dorsal cortex (Figure 2B). When compared to the DCX+/Tbr1+ cells in the neocortex, the general distribution of these cells in the lizard was consistent with the homologies proposed by Karten (Karten, 1969; Butler et al., 2011). Indeed, the DVR has been proposed to be homologous to temporal neocortical areas, such as auditory and secondary somatosensory and visual cortices, that in mammals show high numbers of DCX+/Tbr1+ cells. By contrast, the neocortical regions proposed as homologous of the dorsal cortex, that include primary somatosensory and visual cortices as well as the posterior cingulate, retrosplenial, and postrhinal cortices, are largely devoid of DCX+/Tbr1+ cells. Collectively, these data strongly support that layer II DCX+/Tbr1+ cells represent a conserved cell type in amniotes. In addition, although the sauropsids homologs of mammalian MEC and LEC associated circuits are still poorly defined (Rattenborg and Martinez-Gonzalez, 2011; Allen and Fortin, 2013; Abellán et al., 2014), it is tempting to speculate that non-newly generated DCX+/Tbr1+ cells may be involved in a conserved form of structural plasticity selectively associated to higher order areas of non-spatial learning and memory networks in amniotes. At the same time, our data suggests that the presence of DCX+/Tbr1+ cells in DP derivatives may represent a mammalian innovation. This supports the hypothesis of Reiner that the UL are an evolutionary novelty, but in parallel introduces the possibility that this novelty has been produced by re-using (or co-opting) pre-existing cell types. In particular, we propose that in the transition from the stem-amniote to mammals, DP progenitors instead of exiting from the cell cycle after the production of the DL neurons homologs, continued to proliferate by setting up a LP and/or VP developmental program, giving rise to the UL of the NC. Thus, the evolution of the neocortex could be attributed to a spatial to temporal patterning switch involving DP and LP/VP developmental programs. An interesting aspect of this model is that it could reconcile the developmental data supporting the field homology of the primary progenitors, with the striking similarities existing between neurons of the neocortex and LP and VP derivatives of sauropsids. Future studies in other reptilian species will be required to understand if the distribution of DCX+/Tbr1+ cells in *L. Muralis* represents the basal reptilian condition or, as happen in mice, this species simply lack this feature. An important point will be also to define where and when these cells are generated in different tetrapod species. Indeed, previous studies have shown that the VP and LP progenitors give rise to neurons that tangentially migrate to the neocortex in mice (Puelles, 2011; Teissier et al., 2012). Most of these VP/LP derived cells have a transient existence in mice, but we cannot exclude that in other mammalian species some of these cells may persist for longer post-natal periods (Teissier et al., 2010, 2012). Finally, molecular and functional analyses will be necessary to understand if these cells in different amniote species and pallial derivatives actually represent sister cell types. Nonetheless, as we will discuss in the next paragraphs, beside this intriguing cell population the hypothesis of the co-option of the LP/VP developmental program is supported also by other anatomical and developmental data.

## Similarity in gene expression between PC and neocortical layers II/III and a hypothesis of their evolutionary relationships

**Figure 3:**
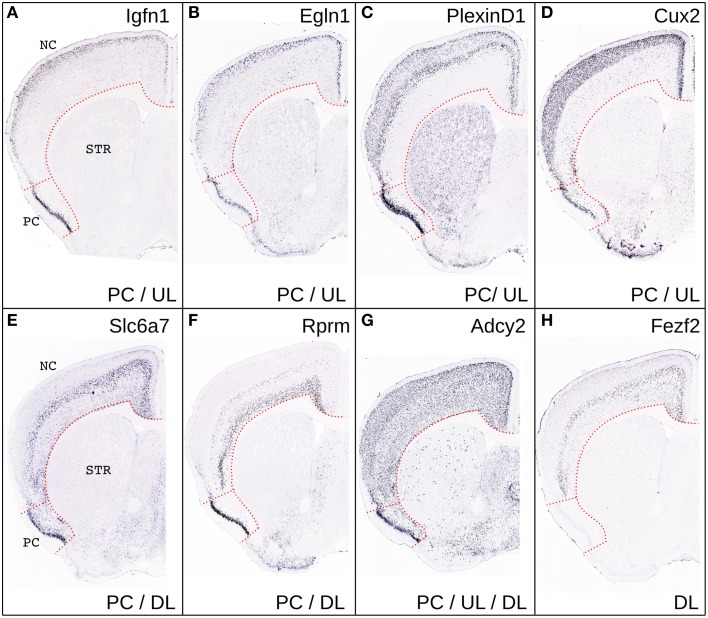
**Comparison of gene expression in PC and NC.** Representative coronal sections from the Allen Brain Atlas showing the expression of few representative genes selectively enriched in the UL and PC **(A–D)**, in DL and PC **(E,F)** in DL, UL and PC **(G)** or only in DL **(H)**. Please note that CUX2, was not present in the list of genes enriched in PC relative to the rest of the gray matter likely because of its relatively widespread expression in the CNS.

**Figure 4:**
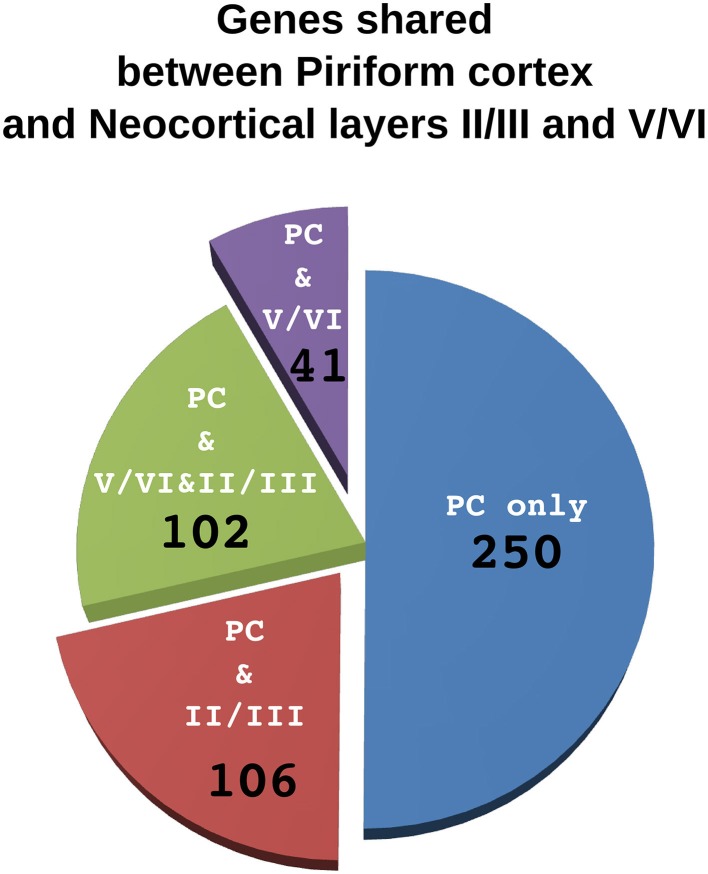
**Comparison of genes shared by piriform cortex and laiers II/III or V/VI of the neocortex.** The pie chart represents the 500 genes enriched in the piriform cortex. Each sector indicate the number of genes shared only with layers V/VI (violet), only with layer II/III (red) or with both layer II/III and V/VI (green). The fraction of genes that are not enriched neither in layer II/III nor in layer V/VI are in blue.

According to our hypothesis, the UL neurons of the neocortex may have sister cell types in other pallial regions. To gain insight on this issue and to identify the best candidate regions whose developmental program may have been co-opted in the evolution of the UL, we performed an analysis in the *in situ* hybridization database of the Mouse Allen Brain Atlas (Lein et al., 2007). In this analysis we compared the lists of the first 500 genes that were enriched relative to the rest of the CNS (contrast structure, gray) in each of the following regions: neocortical layers II/III, layer IV, layer V/VI, piriform cortex, subiculum and cortical subplate (claustro-amygdaloid complex, and endopiriform nucleus; Figure 3, Supplementary data sheet 1A). Layer II/III is closely related to layer IV, with 352 co-expressed genes, and relatively well correlated with layer V/VI, with 250 co-expressed genes (Table 1, Figure 3, Supplementary data sheet 1B, Supplementary Figure 1). Surprisingly, layers II/III cells also shared about 208 enriched genes with the piriform cortex (42%; Table 1, Figures 3, 4, Supplementary Figure 1). The layers V/VI were less related to the piriform cortex with only 143 co-expressed genes (29%; Table 1, Figures 3, 4, Supplementary Figure 1). In addition only 41 genes were exclusively enriched in piriform cortex and layers V/VI but not in layers II/III (29% of PC and layer V/VI common genes). By contrast 106 genes were specifically enriched only in layer II/III and in piriform cortex but not in layer V/VI (51% of PC and layer II/III common genes; Figures 3, 4, Supplementary data sheet 1C). These striking similarities in the gene expression profile raise the intriguing possibility that the developmental program that provided the base for the evolution of the neocortical layers II/III have been that of the olfactory cortex.

**Table 1:** **Percentage of shared genes among the first 500 genes enriched in each of the indicated pallial sub-regions**.

**Percentage of gene co-expression in different pallial regions**						
	**PC**	**NC II/III**	**NC IV**	**NC V/VI**	**C. Sub**	**Subic.**
PC		42	30	29	37	16
NC II/III	42		70	49	21	16
NC IV	30	70		51	13	11
NC V/VI	29	49	51		20	26
C. Sub	37	21	13	20		38
Subic	16	16	11	26	38	

PC, Piriform cortex; NC, Neocortex; C. Sub, Cortical subplate; Subic., Subiculum.

**Figure 5:**
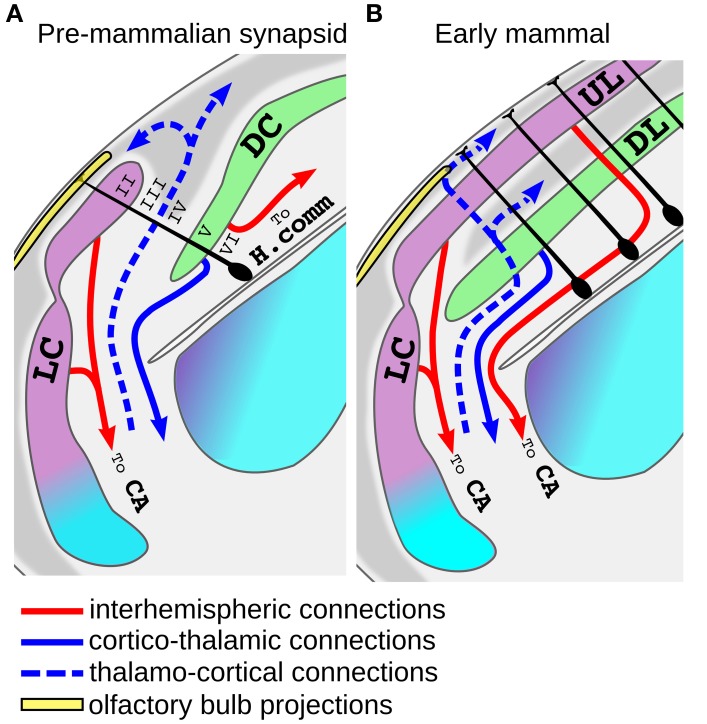
**Model for the origin of the neocortex from the superposition of lateral and dorsal cortex. (A)** Schematic view of the putative organization of the dorso-lateral part of the telencephalon in an early mammaliaform (pre-mammlian synapsid). In these animals the lateral cortex (LC, violet) may have expanded over the dorsal cortex (DC, green), and at some point some radial glial progenitor (dark cells) may have started to produce both LC and DC cells. Note that since the internal anatomy of these animals is unknown, this scheme was based on modern macrosmatic reptiles. Radial glial cells of other brain regions are omitted for clarity. The proposed homology with neocortical layers is indicated with roman nueral. **(B)** Tangential expansion of the progenitors of the proto-neocortical column gave rise to the establishement of the neocortex. Note that the more lateral part of the neocortex maintains a direct olfactory input. Abbreviations: H.comm., hippocampal commissure; AC, Anterior commissure.

This idea is not new and dates back to the beginning of twentieth century. Indeed, early neuroanatomist proposed that a primordium of the neocortex may be found in the superposition of lateral and dorsal cortex, the so called *superpositio lateralis* (Figure 5; Kappers and Theunissen, 1908; Kappers, 1909; De Lange, 1911; Schepers, 1948). This superposition is observed only in some species and its extension correlates with the development of the olfactory system (Ulinsky, 1990). Given that most of the increased encephalization of the first mammaliaformes was due to a huge expansion of the olfactory bulbs and olfactory cortex (Rowe et al., 2011), a substantial development of the lateral superposition could have been present in these species and preceded the emergence of the neocortex. In these superpositions the medial edge of the lateral cortex is located on top of the lateral edge of the dorsal cortex giving rise to a rudimentary six layered arrangement (Figure 5A). Indeed, this region have a dense layer II on top of a sparser layer III that are continuous with the olfactory cortex and receive a direct projection from the olfactory bulb (Minelli, 1967; Regidor, 1977; Desan, 1984; Martinez-Garcia et al., 1991), it posses a parvo-cellular layer IV that receive thalamic inputs (Bruce and Butler, 1984; Desan, 1984; Desfilis et al., 2002) and two deep cellular layers (V and VI) that project to various subcortical targets (Minelli, 1967; Ebner, 1976; Regidor, 1977; Desan, 1984; Hoogland and Vermeulen-Vanderzee, 1989). This layer arrangement is closely reminding that of the neocortex and in particular of the lateral peri-allocortical regions (insular and perirhinal cortices; Figure 2A) which receive olfactory information, lacks layer IV and have a dense layer II that is continuous with the priform cortex (Sanides, 1969; Sanides and Sanides, 1972; Shipley and Geinisman, 1984; Haberly, 1990). Previous authors also highlighted the presence of numerous allocortical features in the peri-allocortical ring, and accordingly proposed that it represent the more ancestral part of the neocortex (Abbie, 1940, 1942; Sanides, 1969; Sanides and Sanides, 1972). The cytoarchitectural similarities between the lateral superposition and the neocortex include also their relationships with bordering regions. In particular, on the medial side the deeper layers of the superposition are in continuity with the non-superposed part of the dorsal cortex while DL of the neocortex are in continuity with the subiculum. An homology between the non-superposed part of the dorsal cortex and the subiculum has been previously proposed (Hoogland and Vermeulen-Vanderzee, 1989). Moreover, this latter region share many features with the DL (Ishizuka, 2001) and accordingly our analyses in the Allen Brain Atlas indicated that it has more enriched genes in common with layer V/VI than layer II/III (26 and 16%, respectively, Table 1, Supplementary Figure 1).

Another interesting aspects of the hypothesis that the neocortex derived from the superposition of lateral and dorsal cortex, is that it may account for some hodological features that the UL shares with the olfactory cortex but not with the reptilian dorsal cortex. For instance, the olfactory cortex of tetrapods possesses homotopic projections to the contralateral hemisphere passing through the anterior commissure (Zeier and Karten, 1973; Butler, 1976; Hoogland and Vermeulen-Vanderzee, 1995; Sassoè-Pognetto et al., 1995; Suárez et al., 2014). Inter-hemispheric projections arising from UL neurons still decussate exclusively through this commissure in monotreme and marsupials, and this is generally thought to represent the basal condition in mammals (Ashwell et al., 1996; Suárez et al., 2014). Nonetheless, in sauropsids inter-hemispheric connections of DP and MP derivatives decussate at the nearby pallial/hippocampal commissure (Butler, 1976; Martinez-Garcia et al., 1990; Atoji et al., 2002). Thus, our hypothesis may account for the strange evolutionary history of the inter-hemispheric connections of the mammalian DP derivatives that at first flipped their direction and coursed a long lateral trip to decussate with fibers of the olfactory cortex at the anterior commissure. Only in eutherian mammals most, but not all, of the neocortical inter-hemispheric connections turned medially again decussating at the corpus callosum (Suárez et al., 2014). Another interesting similarity between the connections of olfactory cortex and UL is that they are both the source of feed-forward projections that flow to a series of hierarchical areas progressively defining sensory objects and ultimately converging on the LEC (Felleman and Van Essen, 1991; Haberly, 2001; Gilbert and Sigman, 2007; Wilson and Sullivan, 2011). In summary, the idea that the six layered neocortex originated from the superposition of lateral and dorsal cortex is consistent with the fossil record and may account not only for the topological position of the neocortex, but also for its basic cytoarchitecthural and hodological features. Unfortunately very little is known about the embryonic development of this putative six-layered primordium in modern reptiles. Guirado and Davila identified radial glial processes crossing both dorsal and lateral cortex in the lateral superposition of the lizard *Podarcis Hispanica* (Guirado and Dávila, 2002) and we made similar observations in Golgi stains of *Lacerta Sicula* (Luzzati unpublished observation). These authors raised the possibility that an independent progenitor domain giving rise to neurons of both dorsal and lateral cortex may actually exist in some living reptiles. In contrast to this interpretation however, Ulinsky reported that during development the layer II of the reptilian dorsal and lateral cortex is a continuous stratum of cells that is secondarily ruptured during differentiation (Ulinsky, 1990). Starting from this latter observation, a possible scenario for the evolution of the neocortex may be that in early mammaliaforms the homologs of UL and DL cells organized in a proto-neocortical column that was initially produced by spatially segregated progenitors. At some point a spatial to temporal patterning switch, together with the evolution of the inside-out neurogenic gradient, led to the generation of the proto-neocortical module from a single population of progenitors (Figure 5A). This crucial event enabled the tangential expansion of this module providing the basis for the establishment of the modern neocortex (Rakic, 1995; Lewitus et al., 2014; Figure 5B). According to the growth rings hypothesis of Sanides, during this tangential expansion the internal parts of the neocortical island progressively lost their allocortical features with the addition of stellate cells in layer IV and a reduction of cell density in layer II (Sanides, 1969; Sanides and Sanides, 1972). An intriguing aspect of this model is that it implies that the early neocortex worked as an higher order association cortex and that primary sensory areas appeared only subsequently. This latter idea has been also recently proposed based on functional models of both mammalian and reptilian allocortices (Fournier et al., 2015).

Several crucial questions remain regarding the emergence of the inside out-gradient of neurogenesis, the appearance of layer IV cells and the arrival of the collo-thalamic projections to the dorsal pallial derivatives.

## Genetic and developmental data supporting a spatial to temporal patterning switch in the evolution of the mammalian neocortex

A hallmark of the evolution of the mammalian neocortex is the emergence of a SVZ in the DP (Martínez-Cerdeño et al., 2006; Cheung et al., 2010), and interestingly the intermediate progenitors (IPc) that populate this germinative layer are mainly involved in the generation of UL neurons (Tarabykin et al., 2001; Martínez-Cerdeño et al., 2006; Kowalczyk et al., 2009). Although, an SVZ is not always evident in sauropsids, studies in turtle and chick showed that putative IP like cells are present in late developmental phases of the LP and VP of turtle and chick (Martínez-Cerdeño et al., 2006; Cheung et al., 2007). The acquisition by DP progenitors of a character (the IPc) that pre-existed in LP/VP progenitors is consistent with our hypothesis. Nonetheless, the IPc step is a common feature in stem cell systems and it has been described for multiple neuronal progenitors populations in both vertebrate and invertebrate brains (Brand and Livesey, 2011). Mammalian DP progenitors may have independently increased the generation of IP to amplify neuron production. Future studies defining the role of the SVZ during pallial development will be necessary to understand the role of this germinative layer in the emergence of the neocortex. While deciphering the developmental program set up by pallial progenitors is a fundamental issue, recent studies also tried to extend previous inter-species comparisons of pallial neuronal types with more modern molecular techniques. The comparison of the chick and mouse transcriptomes of telencephalic regions with either disputed or undisputed homology (Belgard et al., 2013) revealed significant similarities for the hippocampus but failed to identify specific relationships between any other pallial region. The only exception was a weak correlation between the neocortical layer IV and a thalamorecipient field of the nidopallium (a VP derivative). Along with our hypothesis for the evolution of layer II/III it would be interesting to evaluate whether the appearance of stellate cells in layer IV was due to the co-option of the developmental program of the thalamo-recipient VP cells. Unfortunately the olfactory cortex was not analyzed in this study, probably because it is highly reduced in chick. These transcriptomes comparisons supported the view that DP and VP derivatives underwent dramatic changes in morphology and function during amniote evolution (Montiel and Molnár, 2013). At the same time, although such analyses can make a strong case for homology, negative results are more difficult to interpret. Huge differences in the transcriptome do not rule out the occurrence of homologous cell types that greatly changed their relative proportions or mixed with novel cell types. This further indicates the importance of defining the evolutionary history of individual pallial cell types (the so called cell type homology or deep-homology; Arendt, 2008; Shubin et al., 2009) to understand the divergence of DP derivatives in amniotes.

In this perspective, in the last years different authors have analyzed the pattern of expression of the sauropsid orthologs of genes expressed in specific neocortical layers (Nomura et al., 2008, 2013; Dugas-Ford et al., 2012; Suzuki et al., 2012; Chen et al., 2013; Suzuki and Hirata, 2014). The drawback of this approach is that the few individual genes that have been analyzed are expressed by multiple cell types not only in the neocortex but also in other brain regions (Medina et al., 2013). Moreover, the layer specificity of some of the markers of upper layer cells have been disputed (Dugas-Ford et al., 2012). Nonetheless, from these studies a general pattern emerged in which the orthologs of DL markers tend to be expressed more medially than those of the UL. These latter genes are mostly expressed in LP derivatives such as the mesopallium/pallial thickening or the olfactory cortex (Dugas-Ford et al., 2012; Suzuki and Hirata, 2013; Nomura et al., 2014). Since clonal analyses in chick indicate that pallial neurons expressing the orthologs of DL and UL markers are produced by spatially segregated progenitors (Suzuki et al., 2012), these observations are consistent with the hypothesis that the evolution of the mammalian neocortex involved a spatial to temporal patterning switch (Dugas-Ford et al., 2012; Suzuki and Hirata, 2013; Nomura et al., 2014).

Surprisingly, early dorso-medial and dorso-lateral progenitors of the chick pallium were able to sequentially produce cells expressing DL and UL markers *in vitro* (Suzuki et al., 2012). Caution should be made in the interpretation of these data, first because the authors did not verified the purity of the explanted regions and second because the expression of few marker is a very weak evidence that chick and neocortical progenitors generates the same cell types.

Nonetheless, these results introduce the intriguing possibility that an intrinsic temporal patterning mechanism specifying pallio-fugal, thalamo-recipient, and pallio-pallial neuronal types was present in pallial progenitors of the common ancestor of all amniotes or even vertebrates. This idea would be consistent with the fact that temporal patterning of primary progenitors is a major mechanism for generating neuronal diversity in Drosophila (Li et al., 2013a,b; Eroglu et al., 2014). At some point in vertebrate evolution, spatial patterning cues may have differentially repressed specific parts of this program along medio-lateral and anterior-posterior axes. The molecular mechanism that led to the evolution of the six-layered neocortex could thus be a de-repression of the ancestral developmental program in DP progenitors or a subpopulation of them. A similar idea has also been proposed by Luis Puelles to explain the stratified birth dates of VP derived neurons migrating to the neocortex (Puelles, 2011): “One wonders whether this implies a normally repressed, cryptic 6-layer potency existing throughout the pallium, which is simply de-repressed and thus allowed to emerge at the neocortex.” Interestingly, the transcription factor zbtb20 has been recently shown to play a general repressive activity over the specification of neocortical cell types of both UL and DL (Nielsen et al., 2014). In the mammalian pallium, this transcription factor is expressed in MP, LP, and VP but not DP regions and gain and loss of functions have been shown to shift the neocortical limit, at least medially (Nielsen et al., 2007, 2014; Rosenthal et al., 2012). Detailed comparative analyses will be necessary to understand if down-regulation of zbtb20 or other transcriptional repressors in DP progenitors may have played a role in the evolution of the neocortex.

In conclusion, our understanding of the genetic logic of cell type specification in the neocortex and other pallial regions of amniotes is constantly growing and this will likely enable to test current theories of the evolution of the mammalian pallium. These analyses would be greatly helped by the comparison of the genetic fingerprint of more restricted cell populations and the layer II DCX+/Tbr1+ cells represent an attractive candidate for such analyses.

### Conflict of interest statement

The author declares that the research was conducted in the absence of any commercial or financial relationships that could be construed as a potential conflict of interest.

## Supplementary material

Supplementary data sheet 1 **Gene expression comparisons between different pallial regions of the mouse brain. (A)** List of the first 500 genes enriched in neocortical layer II/III, neocortical layer IV, neocortical layer V/VI, piriform cortex, cortical subplate, and subiculum in respect to the rest of the gray matter (contrast structure gray). For each gene the fold change in respect to gray matter calculated by the allen brain atlas is indicated. **(B)** Percentage of sharing, number and lists of genes shared by the selected pallial regions. **(C)** Number and Lists of genes selectively shared by Piriform cortex (PC) and neocortical layers V/VI and by PC and neocortical layers II/III. For each gene a manually evaluated estimate of the level of preferential labeling is indicated by crosses ranging from 0 (low specificity) to 4 (high specificity). A color code is additionally used to indicate high specificity (yellow) medium to low specificity (pink) or absence of any evident specificity (blue).
