# Supplementary figures and images for: A hypothesis for the evolution of the upper layers of the neocortex through co-option of the olfactory cortex developmental program
Source: Front Neurosci. 2015 May 12;9:162. doi: 10.3389/fnins.2015.00162 (PMC4429232; doi:10.3389/fnins.2015.00162)

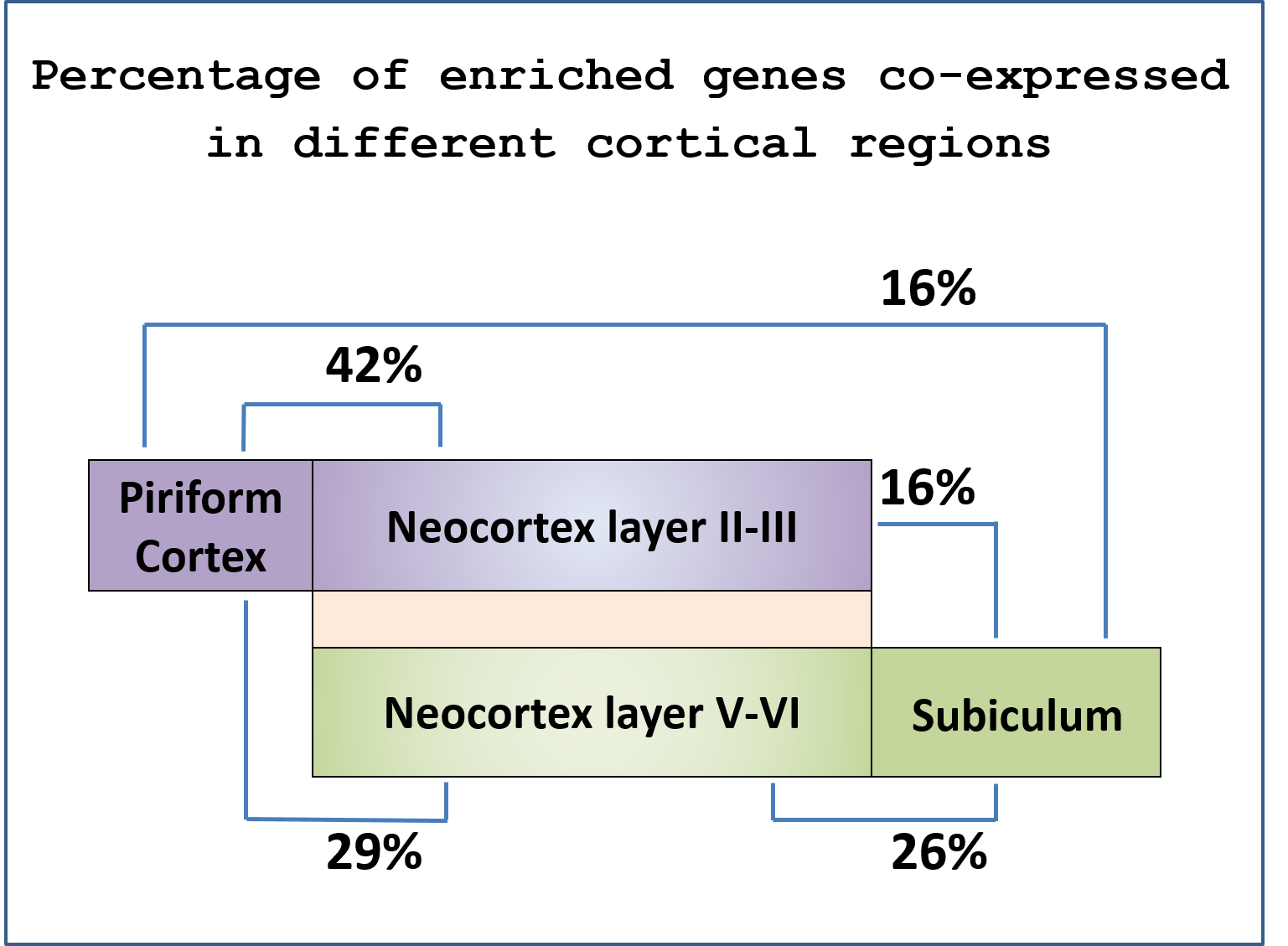

Supplement: Supplementary file 2 [file Image1.TIF]
